# The role of the ventromedial prefrontal cortex in automatic formation of impression and reflected impression

**DOI:** 10.1002/hbm.24996

**Published:** 2020-04-17

**Authors:** Ayahito Ito, Kazuki Yoshida, Kenta Takeda, Daisuke Sawamura, Yui Murakami, Ai Hasegawa, Shinya Sakai, Keise Izuma

**Affiliations:** ^1^ Department of Psychology University of Southampton Southampton United Kingdom; ^2^ Japan Society for the Promotion of Science Tokyo Japan; ^3^ Faculty of Health Sciences Hokkaido University Sapporo Japan; ^4^ Research Institute for Future Design Kochi University of Technology Kochi Japan; ^5^ Department of Rehabilitation for the Movement Functions National Rehabilitation Center for Persons with Disabilities Tokorozawa Japan; ^6^ Department of Occupational Therapy Hokkaido Bunkyo University Hokkaido Japan; ^7^ Graduate School of Health Sciences Hokkaido University Hokkaido Japan; ^8^ School of Economics and Management Kochi University of Technology Kochi Japan

**Keywords:** face, impression formation, interpersonal perception, reflected appraisal, speed dating, ventromedial prefrontal cortex

## Abstract

Previous neuroimaging studies demonstrated that ventromedial prefrontal cortex (vmPFC) activity reflects how much an individual positively views each person (impression). Here, we investigated whether the degree to which individuals think others positively view them (reflected impression) is similarly tracked by activity in the vmPFC by using fMRI and speed‐dating events. We also examined whether activity of the vmPFC in response to the faces of others would predict the impression formed through direct interactions with them. The task consisted of three sessions: pre‐speed‐dating fMRI, speed‐dating events, and post‐speed‐dating fMRI (not reported here). During the pre‐speed‐dating fMRI, each participant passively viewed the faces of others whom they would meet in the subsequent speed‐dating events. After the fMRI, they rated the impression and reflected impression of each face. During the speed‐dating events, the participants had 3‐min conversations with partners whose faces were presented during the fMRI task, and they were asked to choose the partners whom they preferred at the end of the events. The results revealed that the value of both the impression and reflected impression were automatically represented in the vmPFC. However, the impression fully mediated the link between the reflected impression and vmPFC activity. These results highlight a close link between reflected appraisal and impression formation and provide important insights into neural and psychological models of how the reflected impression is formed in the human brain.

## Graphical abstract

We investigated whether the degree to which individuals think others positively view them (reflected impression) is similarly tracked by activity in the vmPFC by using fMRI and speed‐dating events. The results revealed that the value of both the impression and reflected impression were automatically represented in the vmPFC but the impression fully mediated the link between the reflected impression and vmPFC activity. These results highlight a close link between reflected appraisal and impression formation.

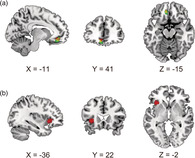

## 1 INTRODUCTION

In modern society, we encounter countless people in our everyday lives. We not only form impressions of the people whom we encounter (Ambady & Rosenthal, 1993; Todorov, Pakrashi, & Oosterhof, 2009; Willis & Todorov, 2006) but also often try to see ourselves from the perspectives of others (i.e., how I think others think of me, a process called “meta‐perception” or “reflected appraisal”) (Cooley, 1902; Kinch, 1963; Mead, 2015). Psychological research has demonstrated that we can recognize how others view us to some extent when we directly interact with them (Carlson & Kenny, 2012; Kenny, 1994; Kenny & DePaulo, 1993; Tagiuri, Blake, & Bruner, 1953), and it also suggests that we use the information to control others' impression of the self (i.e., impression management) (Stopfer, Egloff, Nestler, & Back, 2014). These findings indicate that impression formation and meta‐perception are essential social cognitive abilities that humans possess.

Previous neuroimaging literature has shown that value‐related regions, including the ventromedial prefrontal cortex (vmPFC) and ventral striatum, represent impressions of others in a parametric fashion (Cloutier, Heatherton, Whalen, & Kelley, 2008; Cooper, Dunne, Furey, & O'Doherty, 2012; Ishai, 2007; Ito et al., 2016; Kim, Adolphs, O'Doherty, & Shimojo, 2007; Kranz & Ishai, 2006; Kuhn & Gallinat, 2012; Lebreton, Jorge, Michel, Thirion, & Pessiglione, 2009; Murakami et al., 2018; O'Doherty et al., 2003; Tsukiura & Cabeza, 2011). Evidence further suggests that these regions process impressions of others even when participants are not asked to think about how attractive or pleasant they felt about each target face (Ito et al., 2015; Kim et al., 2007; Lebreton et al., 2009). Lebreton et al. (2009) called the network of these brain regions an “automatic valuation system.” However, it remains unclear whether these regions automatically track the extent to which an individual thinks others like her/him (hereafter referred to as “reflected impression”) in a parametric fashion as well as the extent to which s/he likes them (i.e., impression). Although some previous neuroimaging studies have employed experimental paradigms that require participants to think of how the partner sees the participants (Pfeifer et al., 2009; Powers, Somerville, Kelley, & Heatherton, 2013; Will, Rutledge, Moutoussis, & Dolan, 2017), no study has focused on the neural mechanisms associated with the automatic coding of the reflected impression made just from facial information.

Here, we specifically investigated whether the reflected impression (i.e., the extent to which an individual thinks others like her/him) is automatically represented in the vmPFC and ventral striatum when the faces of others are presented. Previous research has shown that these regions are involved in representing subjective pleasantness induced by positive evaluations by others (Davey, Allen, Harrison, Dwyer, & Yucel, 2010; Ito et al., 2011; Izuma, Saito, & Sadato, 2008; Kawasaki et al., 2016; Meshi, Morawetz, & Heekeren, 2013; Moor, van Leijenhorst, Rombouts, Crone, & Van der Molen, 2010). For example, a previous study showed that the vmPFC is associated with positive feedback about the self (Moor et al., 2010) and with updating self‐value by a social feedback prediction error (Will et al., 2017; Yoon, Somerville, & Kim, 2018). Notably, activity in these regions increases when subjects believe they are liked by others (Davey et al., 2010) and when they expect future social rewards such as positive facial expressions (Spreckelmeyer et al., 2009). These findings suggest that the mere belief that we are liked by others or that we will gain a positive reputation in the future has positive subjective value and that this value is represented in the vmPFC and ventral striatum (Ruff & Fehr, 2014). Taken together with previous findings of impression formation, there is a possibility that reflected impressions and impressions have a link via these regions.

In fact, the psychological literature has demonstrated that impressions affect reflected impressions (i.e., if I like him, I tend to think that he likes me) (Kenny & Albright, 1987; Tagiuri et al., 1953). Tagiuri et al. (1953) studied members attending group meetings and asked them to indicate others whom they liked (i.e., impression) and to guess others who liked them (i.e., reflected impression). The results revealed that the members tended to guess other members who they like would like them, suggesting that the members perceive others' feelings in accordance with their feeling of them. This causal effect is called “congruency” (Tagiuri et al., 1953) or “congruence” (Kenny & Albright, 1987) and implies a critical role of the impression on the formation of the reflected impression. Thus, we hypothesized that, if the same region within the vmPFC is related to both the impression and reflected impression, the link between the reflected impression and vmPFC activity could be explained by the impression.

In the present study, inside an fMRI scanner, participants performed a passive face‐viewing task where they were presented with the faces of people of the opposite gender whom they would meet during subsequent speed‐dating events. We reason that because participants knew that they would meet these individuals in the subsequent speed‐dating events, when they were presented with each face image, the brain would automatically process each face in terms of whether they would be liked by each of them and by whether they would like each of them.

We also investigated whether the brain activity collected in the fMRI session would predict the extent of the impressions and reflected impressions that are formed through direct conversation in the subsequent speed‐dating events. With a deeper understanding of reward‐related regions, researchers have been trying to predict human behavior (i.e., future preferential choices) based on activities in these brain regions (Knutson, Rick, Wimmer, Prelec, & Loewenstein, 2007). Such an approach is often called the brain‐as‐predictor approach (Berkman & Falk, 2013), and a few studies have shown that brain activity forecasts the future liking of others by combining fMRI and a direct interaction paradigm such as speed dating (Cooper et al., 2012; Zerubavel, Hoffman, Reich, Ochsner, & Bearman, 2018). For example, Zerubavel et al. (2018) showed that the activity of the vmPFC and ventral striatum predicts future impressions, suggesting the utility of brain activity as a precursor of future liking. In the present study, we investigated whether both reflected impressions and impressions could also be forecasted by self‐reports and brain activity data collected beforehand.

## 2 MATERIALS AND METHODS

### 2.1 Subjects

The participants of this experiment consisted of two groups: (a) fMRI participants and (2) speed‐dating only participants. The fMRI group included a total of 43 healthy young volunteers with no history of neurological disease. No pathological findings in the brains of the participants were identified using magnetic resonance imaging (MRI). All participants had normal or corrected‐to‐normal vision and declared that they were heterosexual. These participants attended two fMRI sessions and speed‐dating events as described below. Three participants were excluded from the analysis because they rated all faces uniformly in at least one of the rating tasks after the fMRI scanning. Thus, the present fMRI results are based on the data from the remaining 40 participants (18 females, mean age = 20.33 years [range 20–23]).

The speed‐dating only group included a total of 76 healthy young volunteers (38 females, mean age = 22.03 years [range 20–28]). They participated in only one speed‐dating event and did not participate in the fMRI experiment. They are hereafter called “speed‐dating only participants”. All fMRI and speed‐dating only participants were recruited from three local universities. The participants provided written informed consent in accordance with the Declaration of Helsinki, and the study was approved by the ethics committee of Hokkaido University.

### 2.2 Stimuli

The facial photographs for each of the fMRI and speed‐dating participants were taken in a signup session held a few weeks before the initial fMRI session. The participants were told that the photographs would be presented during an fMRI experiment. The photographs were captured using a Panasonic DMC‐LX2 digital camera with a flash and a resolution of 1,920 × 1,080 pixels. The participants were asked to present a neutral facial expression and to look directly into the camera. All images were subsequently downloaded onto a computer and edited in Adobe Creative Cloud (San Jose, CA) to produce greater uniformity across the photographs. The photographs were also resized to 720 × 540 pixels.

### 2.3 Experimental design

**FIGURE 1:**
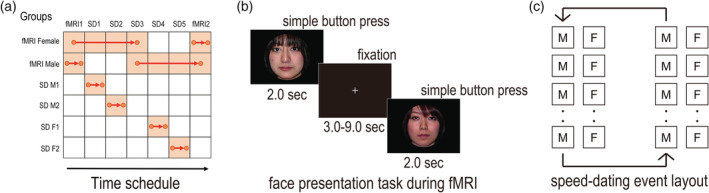
(a) Participant groups and time flow of the present study. The participants were allocated to one of six groups. The female and male participants who were allocated to the fMRI groups (fMRI female and fMRI male) attended fMRI scanning before and after the speed‐dating events. Here, we do not report the results concerning fMRI 2. The female fMRI participants attended the first three speed‐dating events, and the male fMRI participants attended the last three speed‐dating events. Thus, the female fMRI participants had 3‐min talks with the male fMRI participants in the third speed‐dating event. The male participants and female participants who did not participate in the fMRI were allocated to one of the speed‐dating (SD) groups (SD M1, SD M2 or SD F1, SD F2) and attended only one speed‐dating event. (b) During fMRI, the subjects were presented with faces one by one and were asked to press a button as soon as possible when the face image appeared. (c) In the speed‐dating event, the female and male participants sat on a chair facing each other and had a 3‐min talk. After each date, all male or female participants rotated one partner to their left and filled out a questionnaire about the last date

For each fMRI participant, the entire experiment consisted of the following three phases across four separate days: (a) a pre‐speed‐dating fMRI session, (b) three speed‐dating events, and (c) a post‐speed‐dating fMRI session (Figure 1a). Note that since the present study focuses on the neural mechanisms associated with encoding the value of reflected impressions and impressions, the fMRI results from the post‐speed‐dating session will not be reported here.

#### 2.3.1 Pre‐speed‐dating fMRI

During the pre‐speed‐dating fMRI session, inside an fMRI scanner, each participant was presented with the faces of people of the opposite gender whom they would meet in the subsequent speed‐dating events. The female participants were presented with 66 male faces gathered from the fMRI and speed‐dating groups, and the male participants were presented with 63 female faces gathered from the fMRI and speed‐dating participants. The order of presentation of the faces was randomized for each participant. Each face was presented for 2.0 s, and the inter‐stimulus interval, during which a fixation cross was constantly presented, ranged between 3.0 and 9.0 s to maximize the efficiency of the event‐related design (Dale, 1999). The participants were asked to simply press a button as soon as possible when presented with faces because we focused on the neural mechanisms associated with automatic face evaluation (Lebreton et al., 2009). This fMRI task consisted of one run that lasted approximately 8 minutes. In addition, 6‐min resting state scans were collected before the task (the results will not be reported here). After the scanning, the participants were unexpectedly asked to perform five rating tasks where they rated the same faces on the following dimensions: (a) attractiveness, (b) preference, (c) willingness‐to‐talk (WTT), (d) how attractive each person would think that the participant is (reflected attractiveness), and (e) the preference of each person for the participant (reflected preference) (Table 1). These rating tasks were performed using a 7‐point Likert scale (1 = not at all, 7 = very much), and the partner faces were shown one by one in random order. For each rating task, the female participants had 66 trials, and the male participants had 63 trials. The order of the former three rating tasks and the latter two rating tasks was counterbalanced across the participants.

**TABLE 1:** A list of questions used in the pre‐speed‐dating fMRI and speed‐dating event

Pre‐speed‐dating fMRI
1. How attractive is the face? (attractiveness)
2. How much do you like the face? (preference)
3. How much do you want to talk to the person? (willingness‐to‐talk)
4. How attractive would the person think you are? (reflected attractiveness)
5. How much would the person like you? (reflected preference)
Speed‐dating event
1. How was the last date? (date)
2. How attractive was your last partner? (attractiveness)
3. How much do you like your last partner? (preference)
4. How much more do you want to know about the last partner? (know‐more)
5. How attractive does the last partner think you are? (reflected attractiveness)
6. How much do you think the last partner likes you? (reflected preference)
7. How much do you think your last partner wants to know you? (reflected know‐more)
8. Do you know the partner? (friend check)

#### 2.3.2 Speed‐dating event

The fMRI participants attended speed‐dating events several days after the fMRI and talked to the people whose faces were presented during the pre‐speed‐dating fMRI session (Figure 1a). There were a total of five speed‐dating events on three consecutive days (1, 2, and 2 events), and 39–42 participants attended each event. We conducted the five speed‐dating events to ensure plenty of trials in the pre‐speed‐dating fMRI session. The female fMRI participants attended the first three speed‐dating events, and the male fMRI participants attended the last three speed‐dating events. The 76 speed‐dating only participants attended only one speed‐dating event (Figure 1a). The mean duration between the pre‐speed‐dating fMRI scanning session and the participants' first speed‐dating events was 6.12 days (range 1–9 days). One female and one male fMRI participant were absent from one speed‐dating event and the corresponding trials (i.e., faces) and were therefore excluded from analysis of the data from the speed‐dating sessions. All other fMRI participants attended three speed‐dating events.

Each speed‐dating event was held in a large open room and took approximately 3 hours. Upon arrival, each participant received an ID number and a bundle of worksheets (questionnaires). They were asked to take a seat on a chair with the same ID. In each date, the participants had a 3‐min unconstrained conversation with their partners. Following each 3‐min conversation, they were asked to rate their impression of the partner/date (7 questions; see Table 1) using a 7‐point Likert scale (1 = not at all, 7 = very much). The participants were also required to indicate whether they were acquainted with the partner. If the participants indicated that they knew the partner, all the data concerning the partner were excluded from the analysis (mean = 0.40, max = 2). After each date, all male participants or female participants rotated one partner to their left (Figure 1c). At the end of the speed‐dating event, the participants were asked to choose at least half of the partners based on (a) whether they wanted to talk to the partner more (speed‐dating preference choice) and (b) whether they thought the partner wanted to talk to them more (speed‐dating reflected preference choice). All participants were instructed not to reveal any personal information (e.g., name, phone number, or e‐mail address) to partners during the speed‐dating event. Although all participants were led to believe that these speed‐dating events were real (i.e., real opportunities to find a date), in reality, regardless of the outcome of the speed dating, no participant received personal information from any of the partners due to security concerns.

#### 2.3.3 Post‐speed‐dating fMRI session

During the post‐speed‐dating fMRI session, each fMRI participant was presented with the same face pictures presented during the pre‐speed‐dating fMRI session (i.e., the faces of people of the opposite gender whom they had met in the speed‐dating events). The participants underwent the same picture viewing task and another fMRI task, which was inspired by an existing paradigm (Cooper, Dunne, Furey, & O'Doherty, 2014), where they were sequentially presented with (a) a partner's face, (b) their speed‐dating reflected preference choice with regard to the partner, and (c) the partner's speed‐dating preference choices and were asked to rate how much they want to talk to the partner again.

### 2.4 Behavioral analysis

We computed the following four within‐subject correlations to test whether each participant's reflected impression accurately matched the actual perception of each partner: (a) the correlation between the participant's reflected impression ratings (i.e., reflected attractiveness and reflected preference) in the post‐fMRI rating and the partner's impression ratings in the speed‐dating event (i.e., attractiveness and preference); (b) the correlation between the participant's reflected impression ratings in the post‐fMRI rating and the partner's impression ratings in the post‐fMRI rating; (c) the correlation between the participant's reflected impression ratings in the speed‐dating event (i.e., reflected attractiveness, reflected preference, and reflected know‐more) and the partner's impression ratings in the speed‐dating event (i.e., attractiveness, preference, and know‐more); and 4) the correlation between the participant's impression rating in the speed‐dating event and the partner's impression rating in the speed‐dating event.

For statistical analysis, each correlation coefficient was Fisher *z* transformed, and a one‐sample *t* test was conducted. For the fMRI rating scores, we also performed two principal component analyses (PCAs) on an individual basis: one for the three impression ratings (i.e., the attractiveness, preference, and WTT ratings) and the other for the latter two reflected impression ratings (i.e., the reflected attractiveness and reflected preference ratings) (see Table S1 for sample statistics). The first principal component scores from these two PCAs were used for imaging analysis as regressors of interest (see Section 2.7 for details). Other principal component scores were not employed here. Hereafter, we call the first principal component scores from the first PCA for the three impression ratings “impression” and the first principal component scores from the second PCA for the two reflected impression ratings “reflected impression.”

### 2.5 Image acquisition

Whole‐brain imaging was performed using a 3.0‐T MRI scanner (MAGNETOM Prisma, Siemens, Germany) equipped with a 12‐channel head coil array for signal reception. A T2*‐weighted echo planar imaging (EPI) sequence sensitive to blood oxygenation level‐dependent (BOLD) contrast was used for functional imaging with the following parameters: repetition time (TR) = 2,500 ms, echo time (TE) = 30 ms, flip angle = 90°, acquisition matrix = 80 × 80, field of view (FOV) = 240 mm, in‐plane resolution = 3 × 3 mm, number of axial slices = 42, slice thickness = 3 mm, and interslice gap = 0.5 mm. An acquisition sequence tilted at 30° to the intercommissural (anterior commissure‐posterior commissure) line was used to recover magnetic susceptibility‐induced signal losses due to the sinus cavities (Deichmann, Gottfried, Hutton, & Turner, 2003). A high‐resolution (spatial resolution 1 × 1 × 1 mm) structural image was also acquired using a T1‐weighted, magnetization‐prepared rapid‐acquisition gradient echo (MP‐RAGE) pulse sequence. The subject's head motion was restricted using firm padding that surrounded the head. The visual stimuli were presented on a mirror mounted on a head coil through a projector outside the scanner room. The responses were collected using a magnet‐compatible response box. The first four scans were discarded for T1 equilibration effects.

### 2.6 Preprocessing

Data preprocessing and statistical analyses were performed using SPM12 software (Wellcome Department of Imaging Neuroscience, London, UK). All volumes acquired from each subject were realigned to correct for small movements that occurred between scans. This process generated an aligned set of images and a mean image for each subject. The realigned images were subsequently corrected for the different slice acquisition times. Each participant's T1‐weighted structural MRI was coregistered to the mean of the realigned EPI images and segmented to separate the gray matter, which was normalized to the gray matter in a template image based on the Montreal Neurological Institute (MNI) reference brain (resampled voxel size 2 × 2 × 2 mm). Using the parameters from this normalization process, the EPI images were subsequently normalized to the MNI template and smoothed using an 8‐mm full‐width, half‐maximum Gaussian kernel.

### 2.7 Statistical analysis of the imaging data

We employed three general linear models (GLMs) to analyze the fMRI data. GLM 1 contained the impression (principal component scores derived from the principal component analysis for the impression ratings) and the reflected impression (principal component scores derived from the other principal component analysis for the reflected impression ratings) as parametric regressors in this order. Notably, because these two parametric regressors (i.e., principal component scores) are computed from different principal component analyses, they are not orthogonal to each other (see Section 2.4 for details). The mean correlation coefficient between the two principal component scores was 0.39 and significantly larger than 0 (*p* < .01), and these two types of scores have common variance. GLM 2 is the same as GLM 1 except that the order of the two regressors is flipped (i.e., with the reflected impression as the first parametric regressor and the impression as the second parametric regressor). For both GLMs, serial orthogonalization was applied (SPM default) to identify fMRI signals associated with the impression that are not adjusted for the reflected impression (with GLM 1) and fMRI signals associated with the reflected impression that are not adjusted for the impression (with GLM 2) (Mumford, Poline, & Poldrack, 2015). Thus, the results from the first parametric regressor of GLM 1 include impression‐related regions. On the other hand, the results from the *second* parametric regressor of GLM 1 include only regions related to the reflected impression that are adjusted for the impression (i.e., reflected impression‐specific regions) because the second parametric regressor would explain the variance unexplained by the first parametric regressor (Mumford et al., 2015). Similarly, while the results from the first parametric regressor of GLM 2 include reflected impression‐related regions, the results from the *second* parametric regressor of GLM 2 include only regions related to the impression that are adjusted for the reflected impression (i.e., impression‐specific regions). The faces that the subject indicated they were acquainted with were modeled as a regressor of no interest. GLM 3, which models each face separately (average number of regressors = 64.1), was employed to extract the brain activity in response to each face for further ROI analysis.

For all three GLMs, the hemodynamic response to the stimulus onset for each event type was modeled via convolution using a canonical hemodynamic response function. Furthermore, six rigid body motion parameters, 6 temporal derivatives, and 12 quadratic terms were included as regressors of no interest to remove motion‐related artifacts (Friston, Williams, Howard, Frackowiak, & Turner, 1996; Siegel et al., 2014). A high‐pass filter of 1/128 Hz was used to remove low‐frequency noise, and an AR (1) model was used to correct for temporal autocorrelations. For GLM 1 and 2, group‐level random effects analyses were performed by applying one‐sample t‐tests to the first‐level t‐maps. GLM 3 was used to calculate the percentage signal change for mixed‐effect logistic regression analysis explained later, and no group‐level analysis was conducted regarding GLM 3.

### 2.8 ROI definition and statistical threshold

Based on our a priori hypothesis that the vmPFC is involved in encoding the value of both impression and reflected impression, we first performed analysis by restricting the search area within the vmPFC using an anatomical vmPFC mask. The vmPFC mask was created by combining bilateral orbital frontal regions and rectus regions from the anatomical automatic labeling (AAL) atlas (Tzourio‐Mazoyer et al., 2002; Yoon et al., 2018) using WFU PickAtlas (Maldjian, Laurienti, Kraft, & Burdette, 2003). We also conducted a similar analysis by restricting the search area within the ventral striatum using anatomical masks of the bilateral nucleus accumbens from IBASPM 71 atlas (Alemán‐Gómez, Melie‐García, & Valdés‐Hernandez, 2006; Ito et al., 2015). Additionally, we conducted exploratory whole‐brain analysis to test whether any other regions are involved in encoding the value of impressions or reflected impressions. For both analyses, the threshold of significance was set at *p* < .001 at the voxel level (uncorrected for multiple comparisons), with *p* < .05 at the cluster level (FWE corrected for multiple comparisons). The peak voxels of the clusters that exhibited reliable effects are reported in the MNI coordinates. The resulting statistical images are displayed using MRIcroGL (https://www.mccauslandcenter.sc.edu/mricrogl/).

### 2.9 Multilevel mediation analysis

Based on previous findings that revealed that people can form impressions very quickly (Willis & Todorov, 2006) or even unconsciously (Ito et al., 2015), it seems more natural to think that impression formation proceeds to and contributes to the formation of reflected impression formation than vice versa. Thus, we hypothesized that the link between the reflected impression and vmPFC activity would be mediated by the impression that would be formed ahead of the reflected impression. Therefore, we performed multilevel mediation analysis (the independent variable = the first principal component score from the reflected impression ratings about each face; the mediator variable = the first principal component score from the impression ratings about each face; and the outcome variable = the activity of the vmPFC in response to each face). The target cluster of the vmPFC was a common area found in GLM 1 and 2 (see Section 3.2 for details), and vmPFC activity in response to each face was calculated by GLM 3. We used R (https://www.r-project.org/ version 3.5.1), Stan (https://mc-stan.org/ rstan version 2.18.2), and the bmlm package (Vuorre & Bolger, 2018) for this mediation analysis. The bmlm package estimates regression models, with subject‐level and trial‐level parameters estimated simultaneously using Markov chain Monte Carlo procedures (Marks, Copland, Loh, Sunstein, & Sharot, 2018). The prior distributions were zero‐centered Gaussians, with user‐defined standard deviations (defaults to 1,000), and the number of iterations was increased from a default of 2,000 to 10,000 to ensure stable results (Vuorre & Bolger, 2018). Fixed‐effect parameters with the lower and upper limits of a 95% credible interval are shown in each path.

### 2.10 Mixed‐effects logistic regression

To determine whether post‐fMRI ratings and/or brain activity could predict (a) whether the participants wanted to talk to the partner more after the speed‐dating event (speed‐dating preference choice) and (b) whether the participants thought that the partner wanted to talk to them more (speed‐dating reflected preference choice) on an individual level, we ran mixed‐effects logistic regressions. The two types of choices were entered into the separate models as dependent variables, and for each dependent variable, we ran three types of mixed‐effects logistic regressions based on self‐reports, brain activity, and both. Thus, we built six models in total. The first principal component scores from the impression ratings and reflected impression ratings were used as self‐report variables. In addition to the subject number, the gender of the participant was entered as a random intercept. The gender of the partner was not entered because it was always the opposite of the gender of the participant. For the models that used brain activity as independent variables, to avoid the double‐dipping problem (Kriegeskorte, Simmons, Bellgowan, & Baker, 2009), the percentage signal changes were extracted from 10‐mm radius spheres centered in each vmPFC (−3, 48, −6) and ventral striatum (0, 9, −3). These coordinates were identified in a previous study that investigated the role of these regions in predicting the future liking of others (Zerubavel et al., 2018). We used MarsBaR software (http://marsbar.sourceforge.net/) to extract the activity in the ROIs, and the activity was normalized on a within‐subject basis. We also created anatomical masks of the vmPFC and ventral striatum to examine whether the results from the functional ROIs were replicated (see Section 2 for details). Signals from the left and right ventral striatum were averaged and used for analysis in a manner similar to that of the functional ROI analysis.

## 3 RESULTS

### 3.1 Behavioral results

**FIGURE 2:**
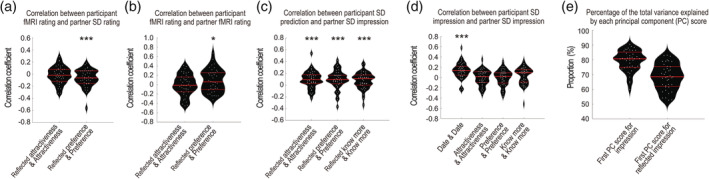
(a) Violin plots of the within‐subject correlations between the fMRI ratings of the fMRI participants and the partner's speed‐dating ratings (left, the participant's post‐fMRI reflected attractiveness rating and the partner's speed‐dating attractiveness rating of the participant; right, the participant's post‐fMRI reflected preference rating and the partner's speed‐dating preference rating of the participant). Each white dot represents the subject's correlation coefficient. (b) Within‐subject correlations between the participant's fMRI ratings and the partner's fMRI ratings (left, the participant's reflected attractiveness rating and the partner's attractiveness rating of the participant; right, the participant's reflected preference rating and the partner's preference rating of the participant). (c) Within‐subject correlations between the participant's (fMRI participants') speed‐dating reflected impression ratings and the partner's speed‐dating impression ratings (from left: the participant's reflected attractiveness rating and the partner's attractiveness rating; the participant's reflected preference rating and the partner's preference rating; the participant's reflected know‐more rating and the partner's know‐more rating). (d) Within‐subject correlations between the participant's (fMRI participants') speed‐dating impression ratings of the partner and the partner's speed‐dating impression ratings of the participant (from left: date, attractiveness, preference, and WTT). (e) Percentage of the variance explained by each principal component on an individual basis (left, percentage of the variance explained by the first principal component score in a PCA for three impression ratings; right, percentage of the variance explained by the first principal component score in a PCA for two reflected impression ratings). SD, speed dating; PC, principal component

Behavioral data are shown in Figure 2a–e. When the participants were given facial information only, they were not very accurate in guessing the partners' impression of the participants themselves, and their reflected attractiveness was not related to the partner's actual impression of their attractiveness (Figure 2a,b); there was no significant correlation between the participants' reflected attractiveness ratings after the fMRI task and the partners' attractiveness ratings in the speed‐dating event (*t*(39) = −0.70, *p* = .49, *d* = −0.11, 95% CI [−0.06, 0.03]) (Figure 2a left). On the other hand, there was a significant negative correlation between the participants' reflected preference ratings and the partners' preference ratings (*t*(39) = −2.75, *p* = .009, *d* = −0.43, 95% CI [−0.11, −0.02]) (Figure 2a right). There was no significant correlation between the participants' reflected attractiveness ratings and the partners' attractiveness ratings after the fMRI task (*t*(39) = −0.12, *p* = .90, *d* = −0.02, 95% CI [−0.08, 0.07]) (Figure 2b left), but there was a significant positive correlation between the participants' reflected preference and the partners' preference (*t*(39) = 2.31, *p* = .03, *d* = 0.36, 95% CI [0.01, 0.18]) (Figure 2b right). Although the correlation between the participants' reflected preference and the partners' preference showed a significant effect, the correlation between the participants' reflected attractiveness and the partners' attractiveness rating showed no significant effect, and the information from a face photograph may be insufficient for a participant to fully predict the partner's impression of her/him.

After the participants interacted with the partners, they became accurate in guessing the partners' impression of the participants themselves, and their reflected impression was linked to the partner's actual impression of them (Figure 2c); there were significant correlations in all three combinations (reflected attractiveness and attractiveness, *t*(39) = 4.03, *p* = .0003, *d* = 0.64, 95% CI [0.05, 0.15]; reflected preference and preference, *t*(39) = 3.51, *p* = .001, *d* = 0.55, 95% CI [0.04, 0.15]; reflected WTT and WTT, *t*(39) = 3.34, *p* = .002, *d* = 0.53, 95% CI [0.03, 0.13]). In addition, there was consensus among the participants regarding how well the date went (*t*(39) = 5.03, *p* = .00001, *d* = 0.80, 95% CI [0.08, 0.19]), but the impression of one another was not reciprocated (attractiveness, *t*(39) = −0.10, *p* = .92, *d* = −0.02, 95% CI [−0.05, 0.046]; preference, *t*(39) = −0.23, *p* = .82, *d* = −0.04, 95% CI [−0.06, 0.04]; WTT, *t*(39) = 1.41, *p* = .17, *d* = 0.22, 95% CI [−0.02, 0.09]) (Figure 2d). These results suggest that a participant and a partner have a certain consensus about the degree of success of speed dating but that the reciprocity of the impression may be independent of the success or failure of the date.

### 3.2 Imaging results

**FIGURE 3:**
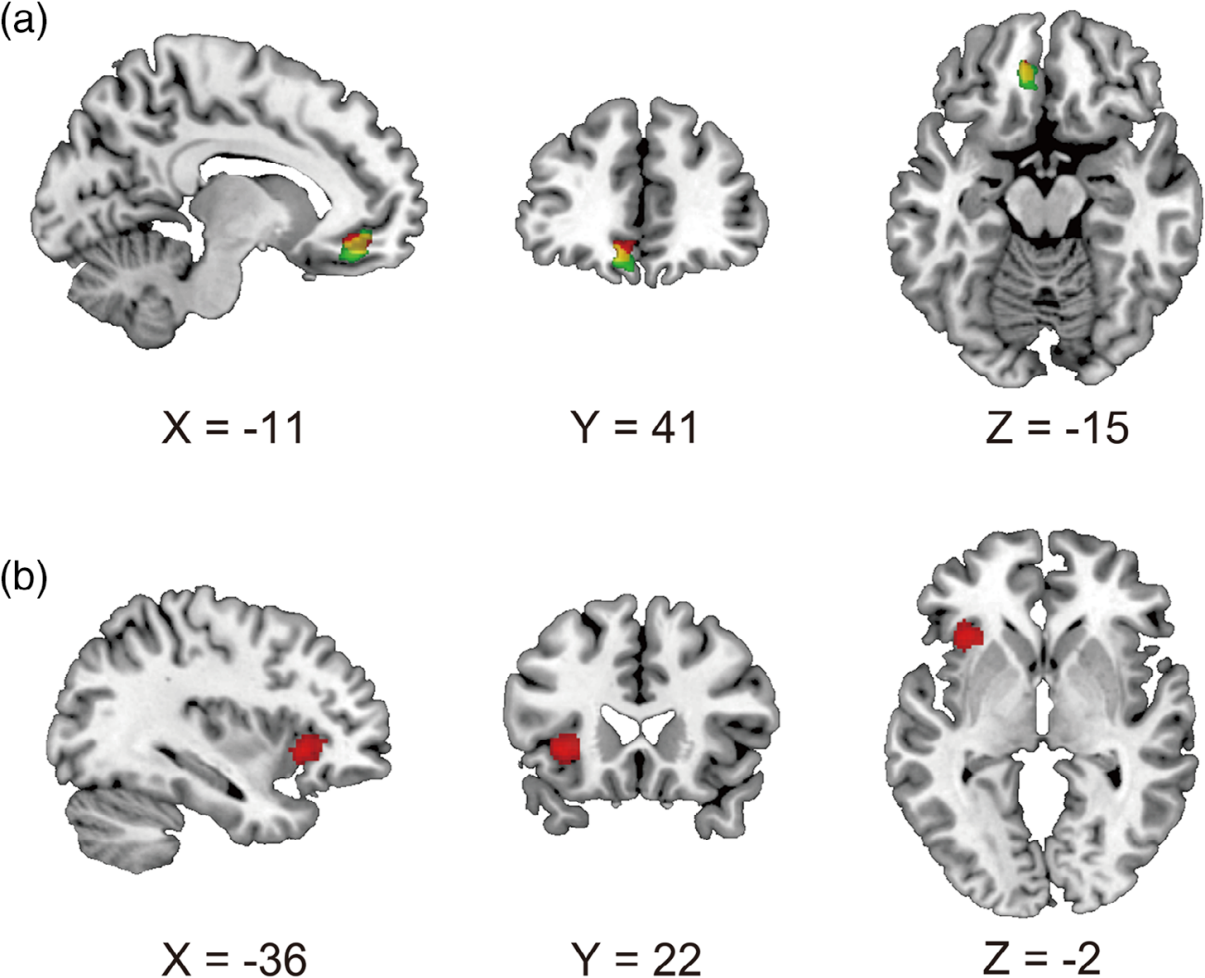
(a) The vmPFC showed a significant positive correlation with the value of both the impression (red; coordinates, −10, 42, −14; *Z* value = 4.16; cluster size = 104) and the reflected impression (green; coordinates, −12, 40, −16; *Z* value = 4.36; cluster size = 61), and a common area was also found (yellow). (b) The left anterior insula showed a significant positive correlation with the value of the impression (coordinates, −36, 22, −2; *Z* value = 4.52; cluster size = 190)

The first parametric regressor of GLM 1 (i.e., impression) with the vmPFC mask revealed significant vmPFC activity (Figure 3a, red; coordinates, −10, 42, −14; *Z* value = 4.16; cluster size = 104). The first parametric regressor of GLM 2 (i.e., reflected impression) with the vmPFC mask also revealed significant vmPFC activity (Figure 3a, green; coordinates, −12, 40, −16; *Z* value = 4.36; cluster size = 61). There was an overlap between these clusters (Figure 3a, yellow), suggesting that mechanisms of impressions and reflected impressions are partially dependent. An exploratory whole‐brain analysis of GLM 1 also revealed that several brain regions, including the bilateral insula, are related to impressions (Table 2, Figure S1A). An exploratory whole‐brain analysis of GLM 2 revealed that the superior frontal gyrus extending to the dorsomedial prefrontal cortex and the PCC as well as the vmPFC are involved in reflected impressions (Table 2, Figure S1B). The analysis also revealed that the left anterior insula has a specific involvement in impressions (i.e., is an impression‐specific region) (Figure 3b). No regions were specifically involved in reflected impressions. We did not find any reflected impression‐specific regions or any significant ventral striatal activity. We also examined whether there are potential gender differences in impression‐related activity and reflected impression‐related activity using two‐sample *t* tests. According to the results, the male participants showed significantly greater impression‐related activity in the bilateral cerebellum (Table 2). We found no other significant clusters that indicate gender differences. These results suggest that there is little impact of gender on vmPFC activity in the present task.

**TABLE 2:** Brain regions showing significant activity in the exploratory whole brain analysis

Region (Brodmann's area)	Coordinates			*Z* value	Cluster size
	*x*	*y*	*z*		
Results of parametric modulation					
Impression‐related regions					
Left anterior insula	−34	24	−2	4.21	222
Left pregenual ACC (24)	−6	38	12	3.91	199
Right parietal cortex (7/40)	26	−54	32	4.48	817
Right anterior insula	36	26	0	4.42	307
Right middle frontal gyrus (44)	38	8	30	4.34	227
Reflected‐impression‐related regions					
Left superior frontal gyrus (9) extending to dorsomedial prefrontal cortex (8)	−14	42	48	4.05	429
Left vmPFC (11)	−12	38	−18	4.54	175
Right PCC (23) extending to left PCC	16	−50	30	4.30	211
Impression‐specific region					
Left anterior insula	−36	22	−2	4.52	190
Reflected‐impression‐specific region					
No suprathreshold activation					
Results of direct comparison between female and male participants					
Impression‐related regions					
Left cerebellum (male > female)	−18	−52	−26	4.49	182
Right cerebellum (male > female)	10	−48	−16	4.47	213
Reflected‐impression‐related regions					
No suprathreshold activation					

Abbreviations: vmPFC, ventromedial prefrontal cortex; ACC, anterior cingulate cortex; MFG, middle frontal gyrus.

### 3.3 The role of the impression as a mediator

**FIGURE 4:**
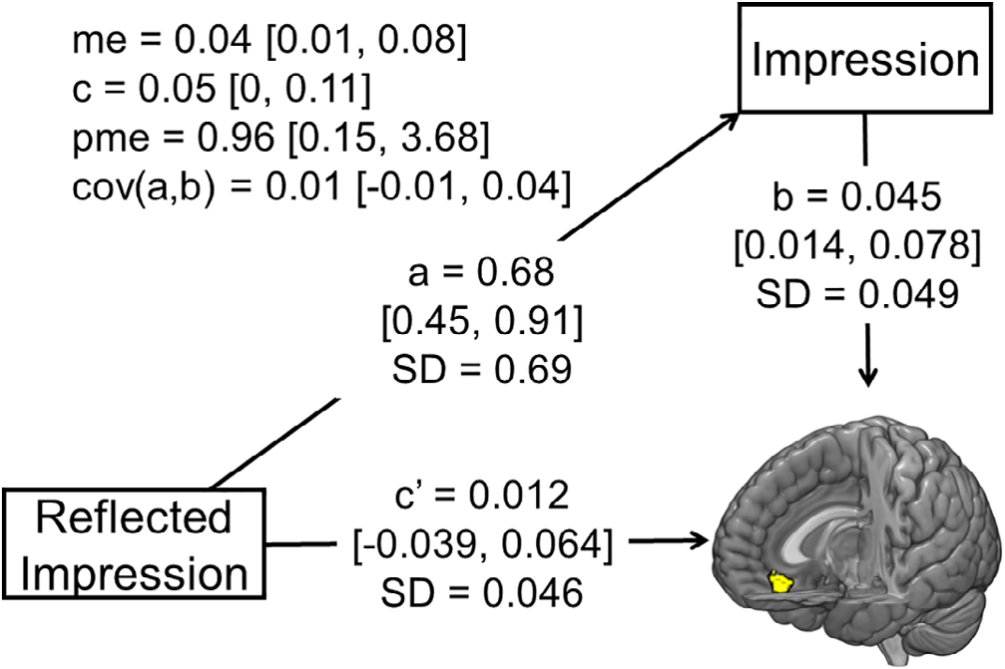
A mediation model revealed that the relationship between the first principal component score from the reflected impression ratings and the activity of the vmPFC was mediated by the first principal component score from the impression ratings. *c* = total effect (direct + indirect effect of the first principal component score from the reflected impression ratings on the activity of the vmpfc), me = mediated effect, *c*′ = direct effect, pme = proportion of the effect that is mediated, cov(a,b) = covariance of trial‐level a and b parameters. The parameters are reported with 95% credible intervals in square brackets

The multilevel mediation analysis showed that the impression mediated the relationship between the reflected impression and vmPFC activity (indirect effect = 0.04, 95% credible interval = [0.01, 0.08]) (Figure 4). A direct effect of the reflected impression on vmPFC activity was diminished after taking the impression into account (*c*′ = 0.012, 95% credible interval = [−0.039, 0.064]), indicating that the impression fully explains the link between the reflected impression and vmPFC activity.

### 3.4 Prediction of choice in the speed‐dating event

The first model regressed the speed‐dating preference choice on the impression and the reflected impression (Table 3). Both self‐report variables significantly positively predicted the choice. The second model regressed the speed‐dating preference choice on the activity of the vmPFC and ventral striatum for each face stimulus. Activity in these regions did not predict the choice. The third model employed both self‐reports and brain activity as independent variables. Only the self‐report variables predicted the choice. Our results did not reveal that brain activity explains future preferential choice. Similar analysis using anatomical masks also revealed no significant effect of brain activity (all *p* values >.1) (see Section 2 for details). In the other three models, only the outcome variable was swapped, and the speed‐dating reflected preference choice was used as the dependent variable. We found results similar to those of the first three models (Table 4) indicating a predominant role of the self‐report variables in predicting future choice.

**TABLE 3:** Logistic regression models predicting speed‐dating preference choice

	Model 1: Self report	Model 2: Brain activity	Model 3: Combined
	Estimate [95% CI]	Estimate [95% CI]	Estimate [95% CI]
Intercept	0.47*** [0.24, 0.71]	0.47*** [0.21, 0.72]	0.47*** [0.24, 0.71]
fMRI actor impression	0.25*** [0.17, 0.33]		0.25***[0.17, 0.33]
fMRI actor reflected impression	0.11* [0.003, 0.23]		0.11* [0.003, 0.23]
vmPFC		−0.01 [−0.12, 0.09]	−0.03 [−0.13, 0.07]
VS		0.09 [−0.02, 0.19]	0.08 [−0.02, 0.18]
Number of observations	2,299	2,299	2,299
AIC	10,416	10,392	10,426
BIC	10,451	10,426	10,472

*Note:* Values inside the square brackets are lower and upper limit of 95% CI.

*** *p* < .01.

* *p* < .05.

**TABLE 4:** Logistic regression models predicting speed‐dating reflected preference choice

	Model 1: Self report	Model 2: Brain activity	Model 3: Combined
	Estimate [95% CI]	Estimate [95% CI]	Estimate [95% CI]
Intercept	0.31*** [0.14, 0.48]	0.31*** [0.13, 0.49]	0.31*** [0.14, 0.48]
fMRI actor impression	0.11** [0.04, 0.17]		0.11**[0.04, 0.17]
fMRI actor reflected impression	0.13* [0.03, 0.23]		0.13* [0.03, 0.23]
vmPFC		−0.02 [−0.11, 0.08]	−0.03 [−0.12, 0.07]
VS		0.02 [−0.08, 0.11]	0.01 [−0.08, 0.11]
Number of observations	2,299	2,299	2,299
AIC	10,001	9,992	10,006
BIC	10,036	10,026	10,051

*Note:* Values inside the square brackets are lower and upper limit of 95% CI.

*** *p* < .001.

** *p* < .01.

* *p* < .05.

## 4 DISCUSSION

In the present study, we investigated the neural correlates of reflected impressions and impressions. Our results showed that both the extent to which we positively view others (i.e., impression) and the extent to which we think others positively view us (i.e., reflected impression) were automatically tracked in common areas within the vmPFC. However, the mediation analysis demonstrated that the impression fully mediated the link between the reflected impression and vmPFC activity. Furthermore, outside of the vmPFC ROI, while the insula activity tracked the participant's impression of faces, there was no region specifically related to the reflected impression.

The results of the parametric modulation analyses and multilevel mediation analysis indicate that the reflected impression does not have unique neural correlates, and they might suggest that while the impression rating likely reflects the subjective value of the face, which is represented in the vmPFC, the reflected impression rating might be constructed based on various deliberate processes, which are likely to be supported by multiple brain regions, rather than simply reflecting the degree of the reflected impression represented in a single brain region. For example, although we attempted to increase the chances for the participants to engage in a reflected appraisal process while viewing faces by instructing them that they would meet these people in subsequent speed‐dating events, there was no objective information that they could use to infer how much each person would like them. The significant positive correlation between the impression and reflected impression ratings suggests that the participants inferred how much they think a person likes them based on their own impression of the person. More specifically, a plausible explanation of the correlational finding is that the participants hoped that they would be liked by those whom they themselves liked (and that they would be disliked by those whom they disliked). This idea is consistent with the previous literature, which has shown a causal relationship where the impression contributes to the formation of the reflected impression in direct social interactions (Elfenbein, Eisenkraft, & Ding, 2009).

One potentially useful psychological model for understanding the triadic relationship among (a) the impression, (b) the reflected impression, and (c) vmPFC activity that we found in the present study (Figure 4) may be the associative‐propositional evaluation (APE) model (Gawronski & Bodenhausen, 2006). This model argues that implicit evaluation reflects automatic activation of mental association, which determines the affective gut reaction to stimuli (e.g., faces), while one's explicit evaluation of stimuli (e.g., impression rating of faces) is formed through propositional reasoning, which validates the information implied by activated association (Gawronski & Bodenhausen, 2006). It seems conceivable to think that when rating faces, the affective reaction to each face directly determines not only its implicit evaluation (which we did not collect in the present study) but also its explicit evaluation (impression rating) (i.e., because there is almost no social desirability bias when rating faces). Thus, the finding that the vmPFC automatically tracked the impression of faces in the present study (Figure 3a) and in previous studies (Ito et al., 2015; Kim et al., 2007; Lebreton et al., 2009) indicates that the vmPFC is a neural locus of this affective gut reaction to facial stimuli. The reflected impression that we asked the participants to rate in this study is likely to require additional mental processes other than propositional processes for impression ratings. As discussed above, we argue that reflected impression ratings were determined based on impression ratings, and affiliation motivation (i.e., the willingness to be liked by especially those who like us) seems to play a key role in determining reflected impression ratings based on impression ratings. This creates an ostensible relationship between vmPFC activity and the reflected impression, which is fully mediated by the impression.

Our behavioral results showed that the subject's prediction of the reflected impression in the fMRI session did not predict the partner's actual appraisal in the speed‐dating event (Figure 2a), indicating that reflected impression ratings formed through observation of the faces of others based on affiliation motivation are generally not accurate and do not accurately reflect the actual impression of others. This result is consistent with the previous literature, which has shown that the first impression formed without any direct interaction with the partner (i.e., zero acquaintance) is inaccurate (Carlson & Kenny, 2012) because the quality of the available data is poor (Kenny, 1994; Kenny & DePaulo, 1993). On the other hand, the 3‐min conversations in the speed‐dating events made the subjects successfully predict the partner's actual appraisal (Figure 2c). This suggests that the accuracy of the reflected impression was improved by employing information acquired though the short social interaction (Kenny, 1994).

Although the subjects successfully predicted the partner's impression of them (Figure 2c), the behavioral results in the speed‐dating events showed no obvious relationship between the participant's impression and the partner's impression (Figure 2d). For example, there was no significant correlation between a participant's preference and a partner's preference for one another (i.e., reciprocal liking). A similar finding is found in previous field research that showed that there is virtually no correlation between how much a participant says she likes her partner and how much he likes her in a romantic context (Walster, Aronson, Abrahams, & Rottmann, 1966). Although there was no obvious reciprocity between the participant's and the partner's impression of one another, they had common sense about the extent to which the date went well, as indicated by the significant correlation between the participant's date ratings and the partner's date ratings (Figure 2d). These findings may suggest that the impression is not affected by the reflected impression and meta‐cognition regarding how well the date went. Previous research has shown that people form impressions rapidly (Ambady & Rosenthal, 1993; Todorov et al., 2009; Willis & Todorov, 2006) and form preferences for faces even when they are not aware of the faces (Ito et al., 2015). Combined with the psychological finding of a causal link between the reflected impression and the impression (Elfenbein et al., 2009; Kenny & Albright, 1987; Tagiuri et al., 1953), the present findings suggest that the impression is formed first and that the reflected impression and meta‐cognition about the date are formed second.

Impression‐related activity of the anterior insula was found after eliminating the effect of the reflected impression. This finding indicates a close link between the anterior insula and impression formation. A recent meta‐analysis focusing on the role of value‐related regions showed functional dissociation between the insula and vmPFC (Bartra, McGuire, & Kable, 2013). Bartra et al. (2013) demonstrated that the vmPFC shows a linear relationship with subjective value, whereas the insula shows a quadratic relationship with subjective value. These patterns suggest that the vmPFC is more involved in the representation of value or positive affect, whereas the insula is more involved in arousal or salience of the target (Uddin, 2015). Because the reflected impression was related to vmPFC activity but not to insula activity, the reflected impression seems to be mainly based on the subjective value of the face (i.e., impression).

Although the present study showed that vmPFC activity reflects the value of reflected impressions, our results do not necessarily exclude the possibility that other regions are related to the formation of reflected impressions during direct communication with others. A brain area that likely plays a pivotal role is the temporoparietal junction (TPJ). The TPJ has been shown to be a key area for mentalizing networks (Schurz, Radua, Aichhorn, Richlan, & Perner, 2014) rather than valuation systems. Previous studies have shown that interactions between the vmPFC and TPJ play important roles in tracking social information (De Martino, O'Doherty, Ray, Bossaerts, & Camerer, 2013; Hare, Camerer, Knoepfle, & Rangel, 2010; Hill et al., 2017; Janowski, Camerer, & Rangel, 2013; Schurz et al., 2014; Smith, Clithero, Boltuck, & Huettel, 2014). Furthermore, Hill et al. (2017) showed that disrupting right TPJ excitability using rTMS prevents reflected appraisal (i.e., how I think others think of me) in a two‐person competitive inspection game. Thus, it seems plausible that the TPJ (and other theory of mind‐related brain regions) plays a key role in reflected impression formation (although it remains uncertain to what extent the TPJ was involved in reflected impression formation in the present study where individuals just passively observed the faces of others). In future research, it would be interesting to test the role of the TPJ in tracking information of the reflected impression using rTMS combined with the speed‐dating paradigm.

The results of our logistic regression analyses revealed that both the self‐reports of the impression and those of the reflected impression in the fMRI session significantly predicted two types of choices in speed dating (Tables 3 and 4): choices based on the subject's preference and choices based on the reflected impression. These results support the present findings indicating a close link between the reflected impression and the impression. However, brain activity did not predict any of the speed‐dating outcome indices. This negative finding is in contrast to a recent fMRI study demonstrating that brain activity significantly predicted a subject's future liking of others after 9 weeks of direct social interaction (Zerubavel et al., 2018). We believe that there is one key difference in the procedures that could potentially explain the discrepancy: the context of the social interaction. Zerubavel et al. (2018) focused on participants' impressions of partners in a nonromantic context where the majority of the participants were female. In contrast, we employed a speed‐dating paradigm (i.e., romantic context) with a relatively equal number of female and male participants. Thus, there is a possibility that implicit concern over expected rejection by a desired person in a romantic context (Montoya, Kershaw, & Prosser, 2018) might affect the activity of the vmPFC and ventral striatum. Furthermore, to the best of our knowledge, among all past neuroimaging studies that took the brain‐as‐predictor approach (for a review, see Knutson & Genevsky, 2018), the present study seems to be the first to report a negative finding of neural data. There might be publication bias (David et al., 2013; Ioannidis, Munafo, Fusar‐Poli, Nosek, & David, 2014; Nissen, Magidson, Gross, & Bergstrom, 2016; Song et al., 2010) or the file drawer problem (Rosenthal, 1979) in this kind of prediction research (e.g., see Lane, Luminet, Nave, & Mikolajczak, 2016).

In conclusion, we showed that the value of the impression and reflected impression were automatically represented in a common area within the vmPFC and that the impression fully mediated the link between the reflected impression and vmPFC activity. These results highlight a close link between impression formation and reflected appraisal and represent an important step toward neural and psychological models of how reflected impressions are formed in the human brain.

## Supporting information


**Figure S1** (a) An exploratory whole‐brain analysis of GLM 1 revealed that several brain regions, including the bilateral insula, pregenual ACC and middle frontal gyrus, are related to impressions. (b) An exploratory whole‐brain analysis of GLM 2 revealed that the superior frontal gyrus extending to the dorsomedial prefrontal cortex, PCC, and vmPFC are involved in reflected impressions.

**Table S1** Example statistics (percentage of variance explained by each principal component [PC]).

## Data Availability

The authors do not have permission to share data due to the restrictions imposed by the administering institution.
