# Supplementary figures and images for: The role of the ventromedial prefrontal cortex in automatic formation of impression and reflected impression
Source: Hum Brain Mapp. 2020 Apr 17;41(11):3045–58. doi: 10.1002/hbm.24996 (PMC7336154; doi:10.1002/hbm.24996)

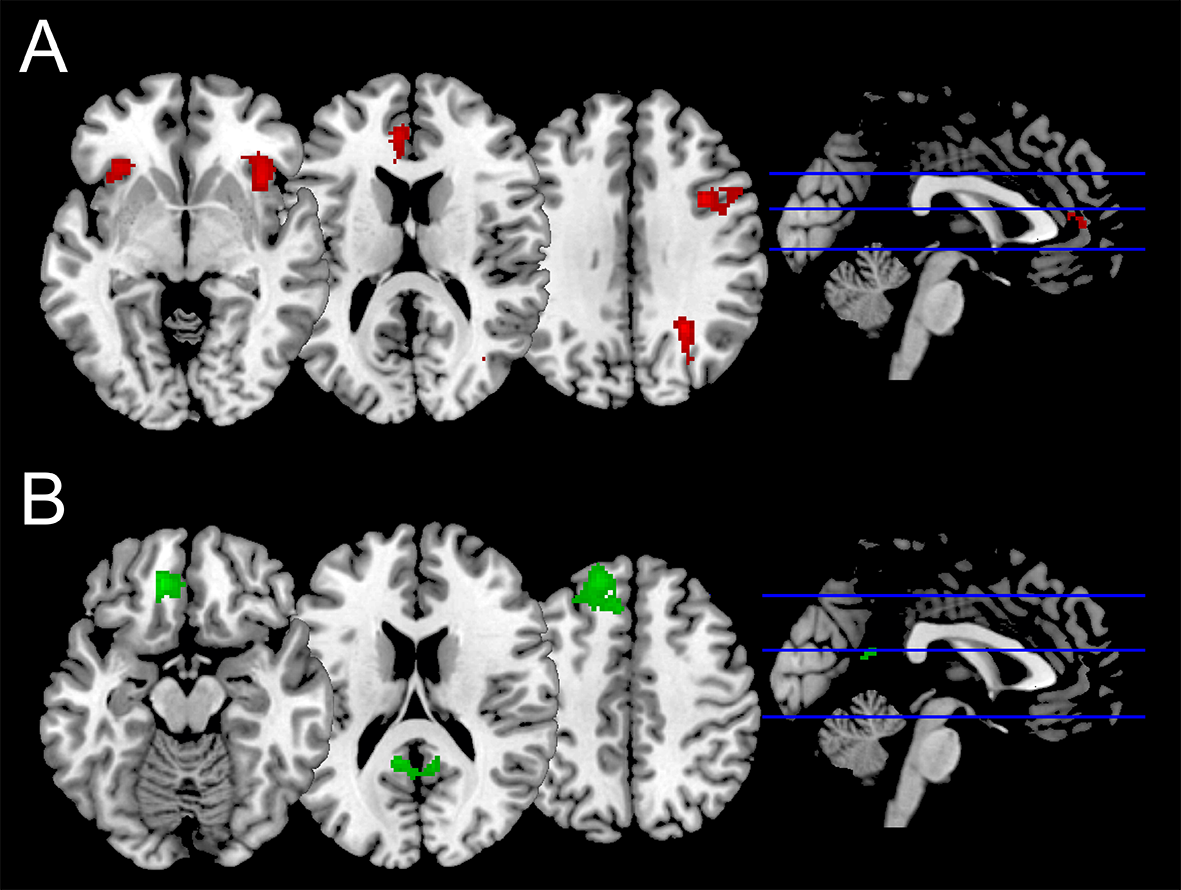

Supplement: Supplementary file 1 — Figure S1 (a) An exploratory whole‐brain analysis of GLM 1 revealed that several brain regions, including the bilateral insula, pregenual ACC and middle frontal gyrus, are related to impressions. (b) An exploratory whole‐brain analysis of GLM 2 revealed that the superior frontal gyrus extending to the dorsomedial prefrontal cortex, PCC, and vmPFC are involved in reflected impressions. [file HBM-41-3045-s001.tif]
