# Supplementary material for: The role of the ventromedial prefrontal cortex in automatic formation of impression and reflected impression
Source: Hum Brain Mapp. 2020 Apr 17;41(11):3045–58. doi: 10.1002/hbm.24996 (PMC7336154; doi:10.1002/hbm.24996)
Supplement: Supplementary file 2 — Table S1 Example statistics (percentage of variance explained by each principal component [PC]). [file HBM-41-3045-s002.docx]

| **Suplementary Table 1 Example statistics (percentage of variance explained by each principal component (PC))** | | | | | |
| --- | --- | --- | --- | --- | --- |
|  | ***Impression*** | | | ***Reflected impression*** | |
|  | **PC1** | **PC2** | **PC3** | **PC1** | **PC2** |
| Participant 1 | 86.59 | 8.74 | 4.67 | 79.37 | 20.63 |
| Participant 3 | 79.75 | 12.49 | 7.77 | 83.09 | 16.91 |
|  | | | |  |  |
